# Supplementary material for: The IKK/NF-κB signaling pathway requires Morgana to drive breast cancer metastasis
Source: Nat Commun. 2017 Nov 21;8:1636. doi: 10.1038/s41467-017-01829-1 (PMC5696377; doi:10.1038/s41467-017-01829-1)
Supplement: Supplementary file 1 — Supplementary Information [file 41467_2017_1829_MOESM1_ESM.pdf]

Supplementary Figure 1

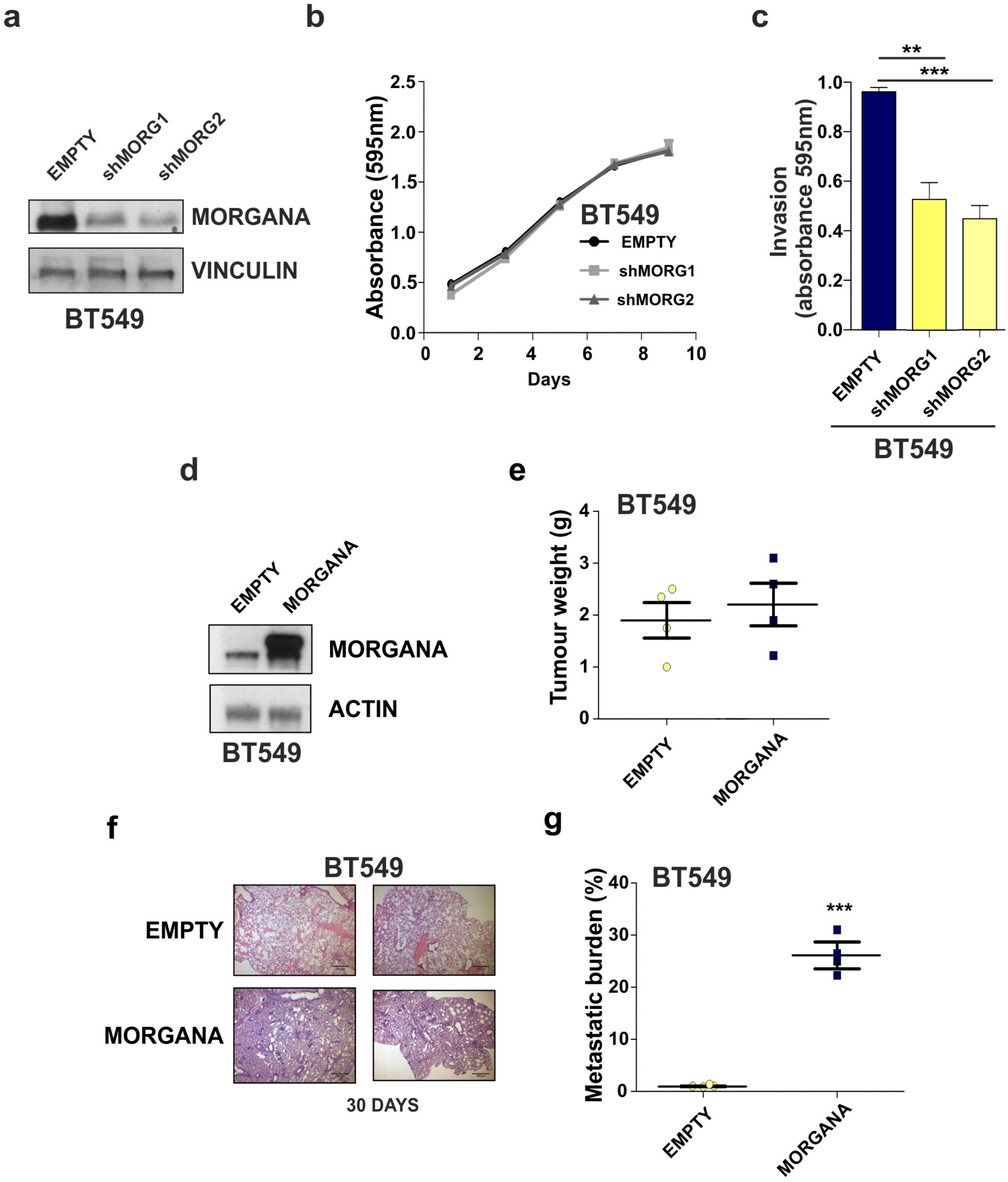

Supplementary Figure 1. Morgana downregulation or overexpression in BT549 influences invasion and metastasis formation. (**a**) Immunoblotting of Morgana and Vinculin in BT549 cells infected with empty vector (EMPTY) or two independent Morgana shRNAs (shMORG1, shMORG2). (**b**) Growth curves of BT549 cells EMPTY or shMORG1 and shMORG2. (**c**) Quantification of invasion assays performed on BT549 EMPTY or shMORG1 and shMORG2. (**d**) Western blot analysis of BT549 infected with an empty vector (EMPTY) or a lentivirus coding for Morgana (MORGANA) immunostained with Morgana and Actin as loading control. (**e**) Tumour weight 30 days after subcutaneous injection of BT549 EMPTY or MORGANA in NSG mice (n=4 NSG mice per group). (**f**,**g**) Representative haematoxylin and eosin stained sections (**f**) and percentage of lung metastatic area (**g**) of mice injected with BT549 EMPTY or MORGANA (n=4 NSG mice per group). Data are the results of at least three independent experiments. Bars in graphs represent standard errors (**$p < 0.01$; ***$p < 0.001$).

Supplementary Figure 2

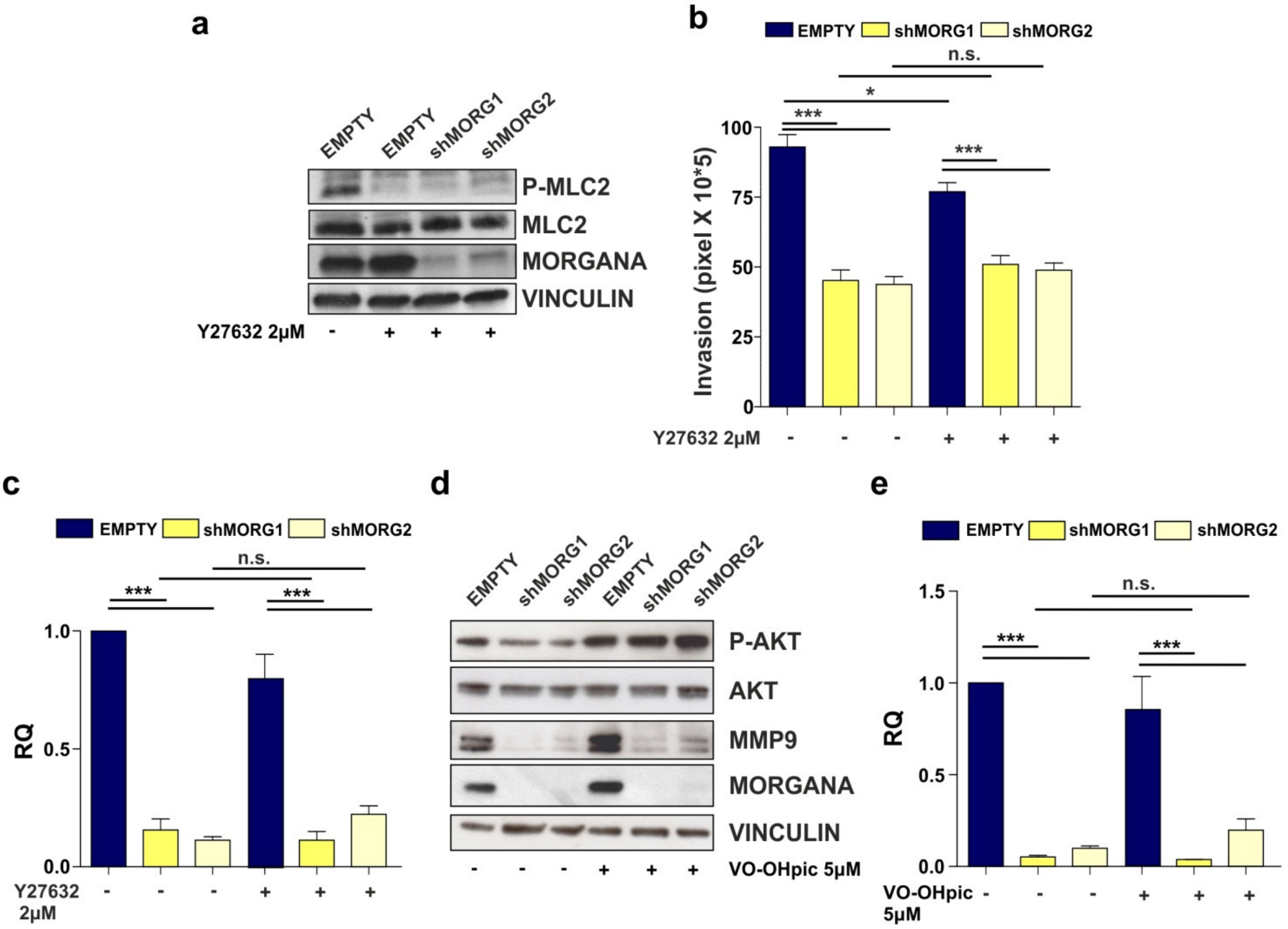

Supplementary Figure 2. Morgana dependent invasion and MMP9 expression do not rely on ROCK/PTEN/AKT pathway. (**a**) Western blot analysis of MDA-MB-231 infected with an empty vector (EMPTY) or two independent shRNAs targeting Morgana (shMORG1, shMORG2) treated or not with the ROCK inhibitor Y27632 (2µM for 30 min) immunostained with P-MLC2, MLC2, Morgana and Vinculin as loading control. (**b**) Transwell matrigel invasion assays on MDA-MB-231 EMPTY or shMORG1 and shMORG2 treated or not with the ROCK inhibitor Y27632 (2µM for 24h). (**c**) Gene expression analysis by Real-time PCR of MMP9 in MDA-MB-231 EMPTY or shMORG1 and shMORG2 untreated or treated with Y-27632 (2µM for 24h). (**d**) Western blot analysis of P-AKT, AKT, MMP9, Morgana and Vinculin on MDA-MB-231 EMPTY or shMORG1 and shMORG2 treated or not with VO-OHpic (5µM for 30 min). (**e**) Gene expression analysis by Real-time PCR of MMP9 in MDA-MB-231 EMPTY or shMORG1 and shMORG2 untreated or treated with VO-OHpic (5µM for 24h). Data are the results of three independent experiments. Bars in graphs represent standard errors (*$p < 0.05$; ***$p < 0.001$).

Supplementary Figure 3

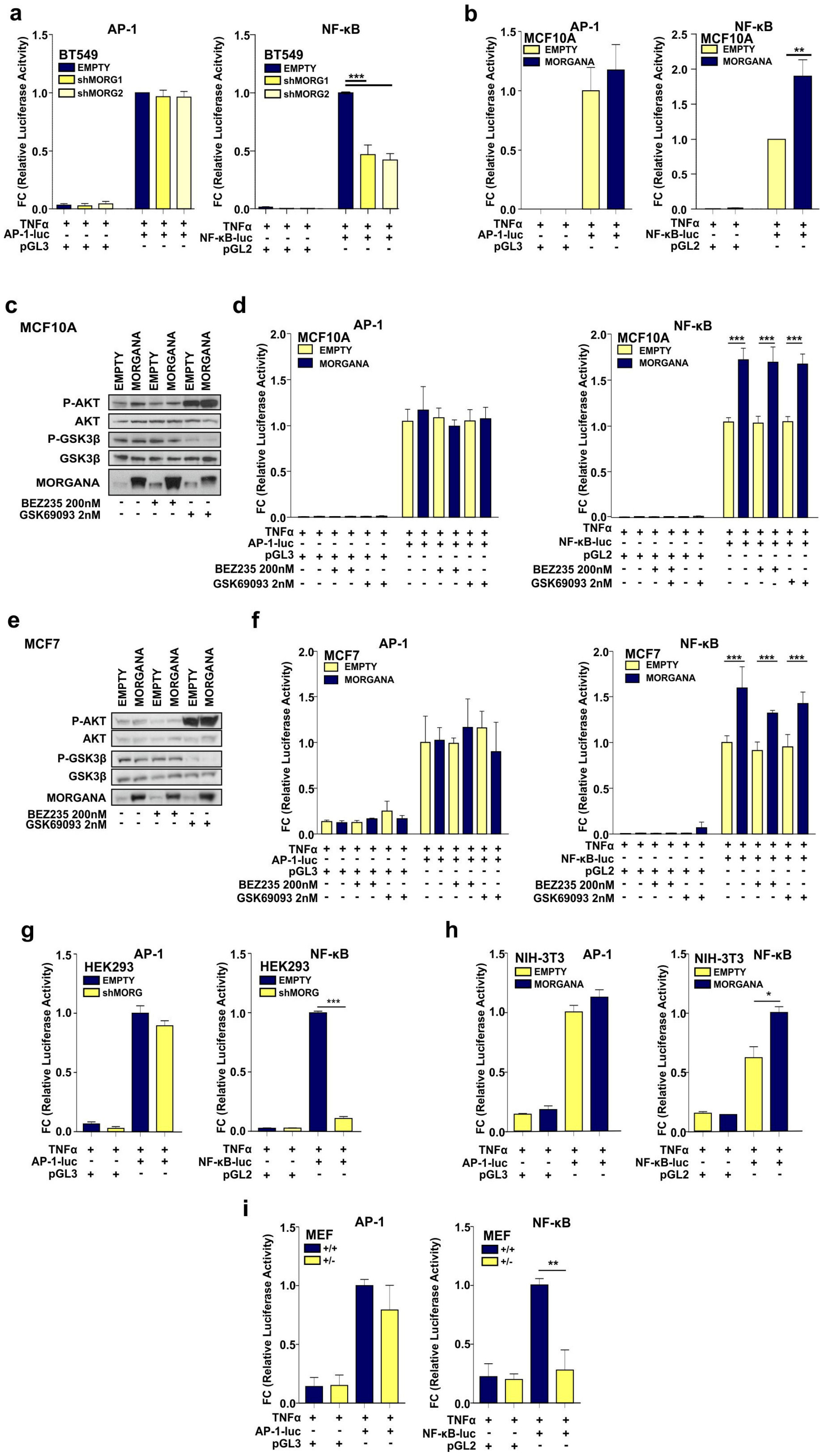

Supplementary Figure 3. Alteration in Morgana expression levels causes a corresponding variation in NF-κB activity. (**a**,**b**) Luciferase assays of AP-1 (left) and NF-κB (right) activity in (**a**) BT549 infected with an empty vector (EMPTY) or shRNAs targeting Morgana (shMORG1 and shMORG2), (**b**) MCF10A infected with an empty vector (EMPTY) or overexpressing Morgana (MORGANA). (**c**) Western blot analysis of MCF10A infected with an empty vector (EMPTY) or overexpressing Morgana (MORGANA) treated or not with the PI3K inhibitor BEZ235 (200nM for 24h) or the AKT inhibitor GSK69093 (2nM for 24h) and immunostained with P-AKT, AKT, P-GSK3β, GSK3β and Morgana. (**d**) Luciferase assays of AP-1 (left) and NF-κB (right) activity assessed in MCF10A EMPTY or MORGANA treated or not with the PI3K or AKT inhibitor. (**e**) Western blot analysis of MCF7 infected with an empty vector (EMPTY) or overexpressing Morgana (MORGANA) treated or not with the PI3K inhibitor BEZ235 (200nM for 24h) or the AKT inhibitor GSK69093 (2nM for 24h) and immunostained with P-AKT, AKT, P-GSK3β, GSK3β and Morgana. (**f**) Luciferase assays of AP-1 (left) and NF-κB (right) activity assessed in MCF7 EMPTY or MORGANA treated or not with the PI3K or AKT inhibitor. (**g-i**) Luciferase assays of AP-1 (left) and NF-κB (right) activity in (**g**) HEK293 infected with an empty vector (EMPTY) or a shRNA targeting Morgana (shMORG), (**h**) NIH-3T3 infected with an empty vector (EMPTY) or overexpressing Morgana (MORGANA) and (**i**) primary mouse embryonic fibroblasts (MEFs) from *morgana* +/- mice and wild type controls. For luciferase assays cells were transfected with an AP-1 or NF-κB luciferase reporter or a control vector. Data are the results of three independent experiments. Bars in graphs represent standard errors (*p < 0.05; **p < 0.01; ***p < 0.001).

Supplementary Figure 4

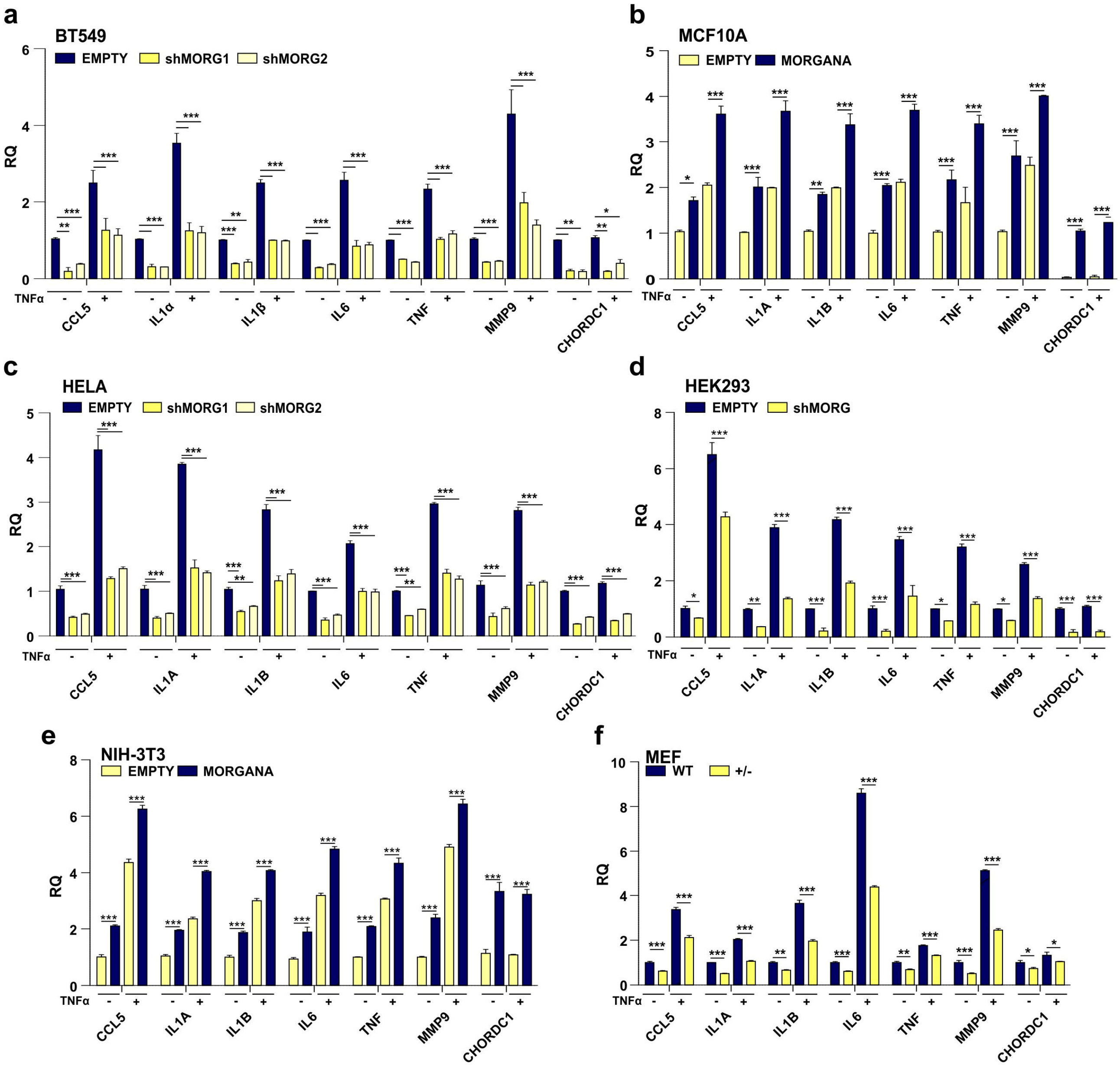

Supplementary Figure 4. Alteration in Morgana expression levels causes a corresponding variation in NF-κB target genes expression. (**a-f**) Gene expression analysis by PCR of NF-κB target genes in (**a**) BT549 infected with an empty vector (EMPTY) or shRNAs targeting Morgana (shMORG1 and shMORG2), (**b**) MCF10A infected with an empty vector (EMPTY) or overexpressing Morgana (MORGANA), (**c**) HeLa infected with an empty vector (EMPTY) or shRNAs targeting Morgana (shMORG1 and shMORG2), (**d**) HEK293 infected with an empty vector (EMPTY) or a shRNA targeting Morgana (shMORG1), (**e**) NIH-3T3 infected with an empty vector (EMPTY) or overexpressing Morgana (MORGANA) and (**e**) primary mouse embryonic fibroblasts (MEFs) from *morgana* +/- mice and wild type controls. Before RNA extraction cells were treated or not with 10nM TNFα for 4h. Data are the results of three independent experiments. Bars in graphs represent standard errors (*p < 0.05; **p < 0.01; ***p < 0.001).

Supplementary Figure 5

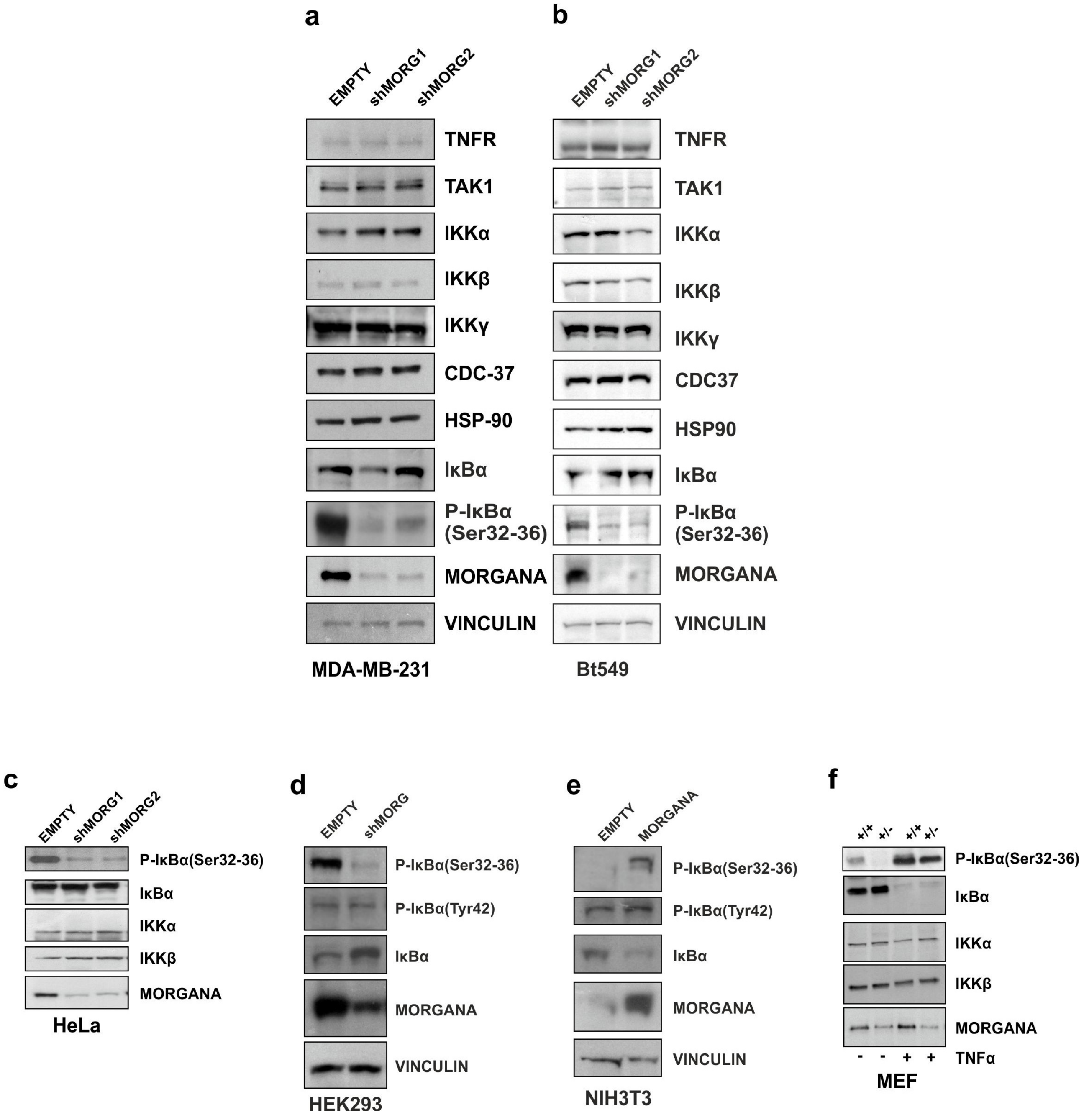

Supplementary Figure 5. Alteration in Morgana expression levels causes a corresponding variation in IκBα phosphorylation independently from PI3K and AKT activation. (**a**,**b**) Western blot analysis of TNF-R1, TAK1, IKKα, IKKβ, IKKγ, CDC37, HSP90, IκBα, P-IκBα(Ser32/36), Morgana and Vinculin on MDA-MB-231 (**a**) or BT549 (**b**) infected with an empty vector (EMPTY) or two independent shRNAs targeting Morgana (shMORG1, shMORG2). (**c**) Western blot of HeLa infected with an empty vector (EMPTY) or two Morgana shRNAs (shMORG1, shMORG2) immunostained for P-IκBα(Ser32/36), IκBα, IKKα, HSP90, IKKβ and Morgana. (**d**) Immunoblot of P-IκBα(Ser32/36), P-IκBα(Tyr42), IκBα, P-IKKα/β, Morgana and Vinculin in HEK293 infected with an empty vector (EMPTY) or depleted for Morgana (shMORG). (**e**) Western blot analysis of P-IκBα(Ser32/36), P-IκBα(Tyr42), IκBα, P-IKKα/β, Morgana and Vinculin on NIH3T3 infected with an empty vector (EMPTY) or a Morgana coding vector (MORGANA). (**f**) Primary mouse embryonic fibroblasts (MEFs) from *morgana* +/- mice and wild type controls immunostained for P-IκBα(Ser32/36), IκBα, IKKα, IKKβ and Morgana treated or not with 10nM TNFα for 15 minutes. Data are the results of three independent experiments.

Supplementary Figure 6

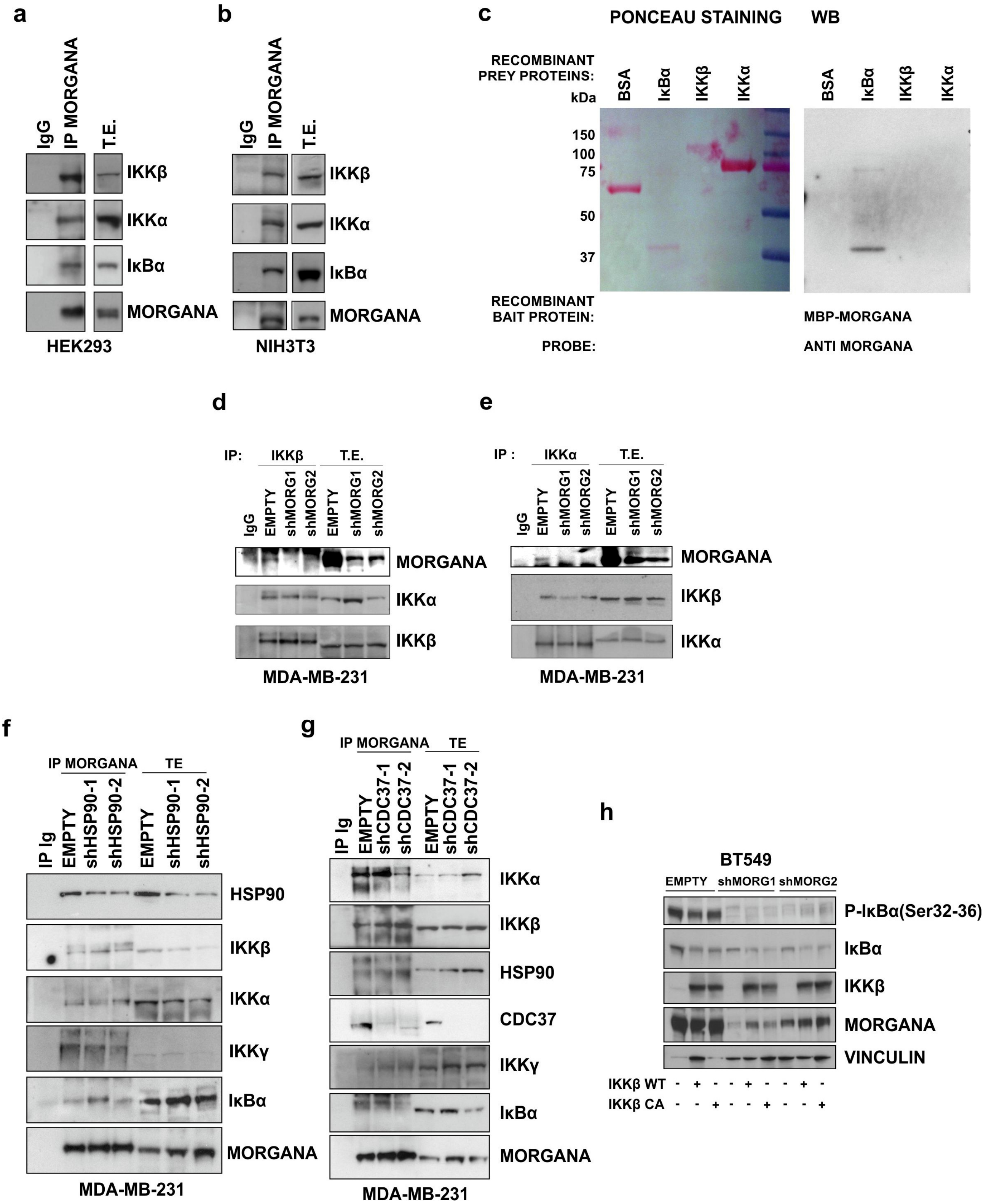

Supplementary Figure 6. Morgana is a component of the IKK complex. (**a**,**b**) Immunoprecipitation of Morgana from HEK293 (**a**) or NIH-3T3 (**b**) immunoblotted with IKKβ, IKKα, IκBα and Morgana. (**c**) Far western blotting. 1 μg of recombinant protein (BSA, IκBα, IKKβ and IKKα) were separated by SDS-page and transferred onto nitrocellulose membrane. Proteins were denatured, renatured and then incubated with 3μg of MBP-Morgana. Left panel: ponceau staining Right panel: membrane was probed with an antibody against Morgana. BSA served as negative control. (**d**) IKKβ was immunoprecipitated from MDA-MB-231 EMPTY or shMORG1, shMORG2 and immunoblotted with antibodies against Morgana, IKKα and IKKβ. (**e**) IKKα was immunoprecipitated from MDA-MB-231 EMPTY or shMORG1, shMORG2 and immunoblotted with antibodies against Morgana, IKKβ and IKKα. (**f**) Immunoprecipitation of Morgana from MDA-MB-231 infected with two different shRNAs targeting HSP90 (shHSP90-1 and shHSP90-2) or a control vector (EMPTY) immunoblotted with HSP90, IKKβ, IKKα, IKKγ, IκBα and Morgana. (**g**) Immunoprecipitation of Morgana from MDA-MB-231 infected with two different shRNAs targeting CDC37 (shCDC37-1 and shCDC37-2) or control vector (EMPTY) immunoblotted with, CDC37, HSP90, IKKβ, IKKα, IKKγ, IκBα and Morgana. (**h**) Western blot analysis of P-IκBα, IκBα, IKKβ, Morgana and Vinculin from total protein extracts of BT549 EMPTY, shMORG1, shMORG2 transfected or not with IKKβ wild type (WT) or constitutively active (CA). Data are the results of three independent experiments.

Supplementary Figure 7

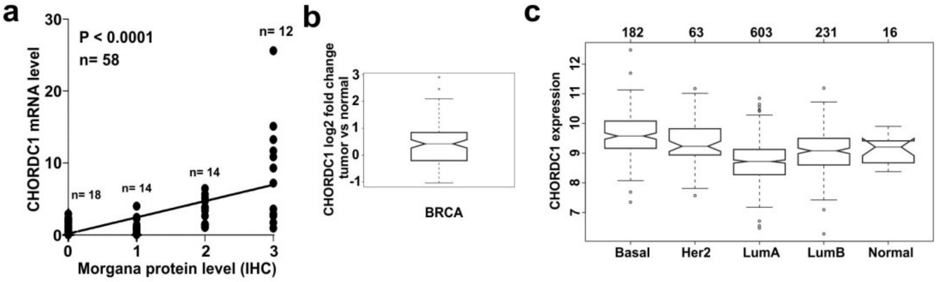

d

Wilcoxon rank sum test p values

	Her2	LumA	LumB
**Basal**	0.01175	2.067e-40	9.964e-14
**Her2**	NA	2.332e-11	0.003454
**LumA**	NA	NA	5.435e-10
**LumB**	NA	NA	NA

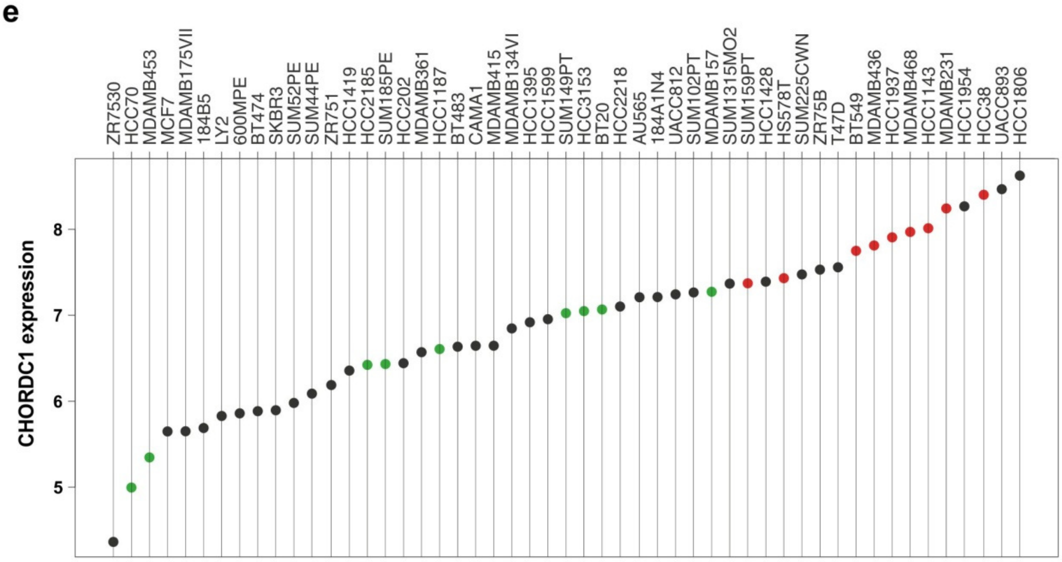

f NF-kB pathway correlation in breast cancer cell lines

All cell lines

GENE SET	FDR.q.val	NES
JAIN_NFKB_SIGNALING	0.0000	2.7
HINATA_NFKB_TARGETS_FIBROBLAST_UP	0.0000	2.3
HINATA_NFKB_IMMU_INF	0.0000	2.1

TNBC cell lines

GENE SET	FDR.q.val	NES
JAIN_NFKB_SIGNALING	0.0000	2.6
HINATA_NFKB_TARGETS_FIBROBLAST_UP	0.0384	1.6

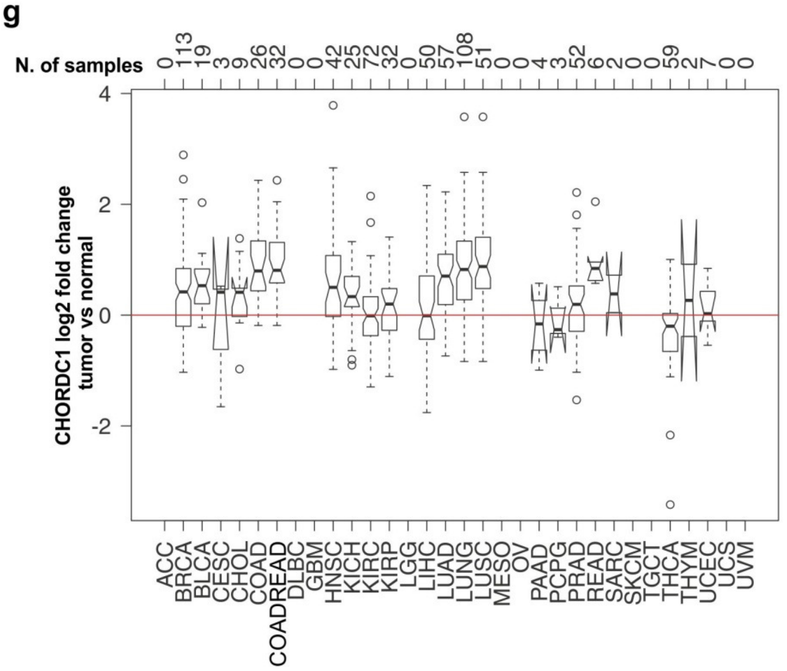

h

p values of CHORDC1 expression in tumor vs normal samples

	Pval	BH
LUNG	1.1e-15	2.3e-14
COADREAD	8.8e-09	7.7e-08
LUAD	1.4e-08	7.7e-08
LUSC	1.3e-08	7.7e-08
BRCA	1.6e-07	7.1e-07
COAD	5.7e-07	2.1e-06
HNSC	1.1e-05	3.4e-05
BLCA	5.3e-05	0.00015
THCA	0.00023	0.00055
KICH	0.014	0.03
READ	0.031	0.063
PRAD	0.091	0.17
CHOL	0.16	0.28
KIRP	0.25	0.39
LIHC	0.45	0.66
SARC	0.5	0.69
UCEC	0.58	0.75
PAAD	0.62	0.76
CESC	1	1
KIRC	0.87	1
PCPG	1	1
THYM	1	1

Supplementary Figure 7. Morgana expression in human breast cancer. (**a**) Spearman correlation between CHORDC1 (Morgana coding gene) mRNA levels and Morgana protein expression levels detected by immunohistochemistry in 58 breast cancer patients from SGBH cohort (Pearson correlation coefficient = 0.6). (**b**) Morgana is significantly up-regulated in tumours compared to adjacent normal tissue. The distribution of the log fold change in the 113 samples is shown. Median logarithmic fold change is 0.419 (P=1.61e-07, Wilcoxon rank-sum test). (**c**) Morgana expression levels in different breast cancer subtypes. (**d**) Table showing Wilcoxon rank sum test p values of graph in **c**. (**e**) CHORDC1 expression levels in breast cancer cell lines. (**f**) Table showing signatures positively correlated with CHORDC1 mRNA levels generated by GSEA analysis of ranked NF-κB gene expression data in all breast cell lines and TNBC cell lines. (**g**) CHORDC1 expression levels in different cancer subtypes present in TCGA dataset for which data on adjacent normal tissue were present. (**h**) Table showing p values of CHORDC1 expression level in tumour versus normal tissues of samples present in graph **g.**

Supplementary Figure 8

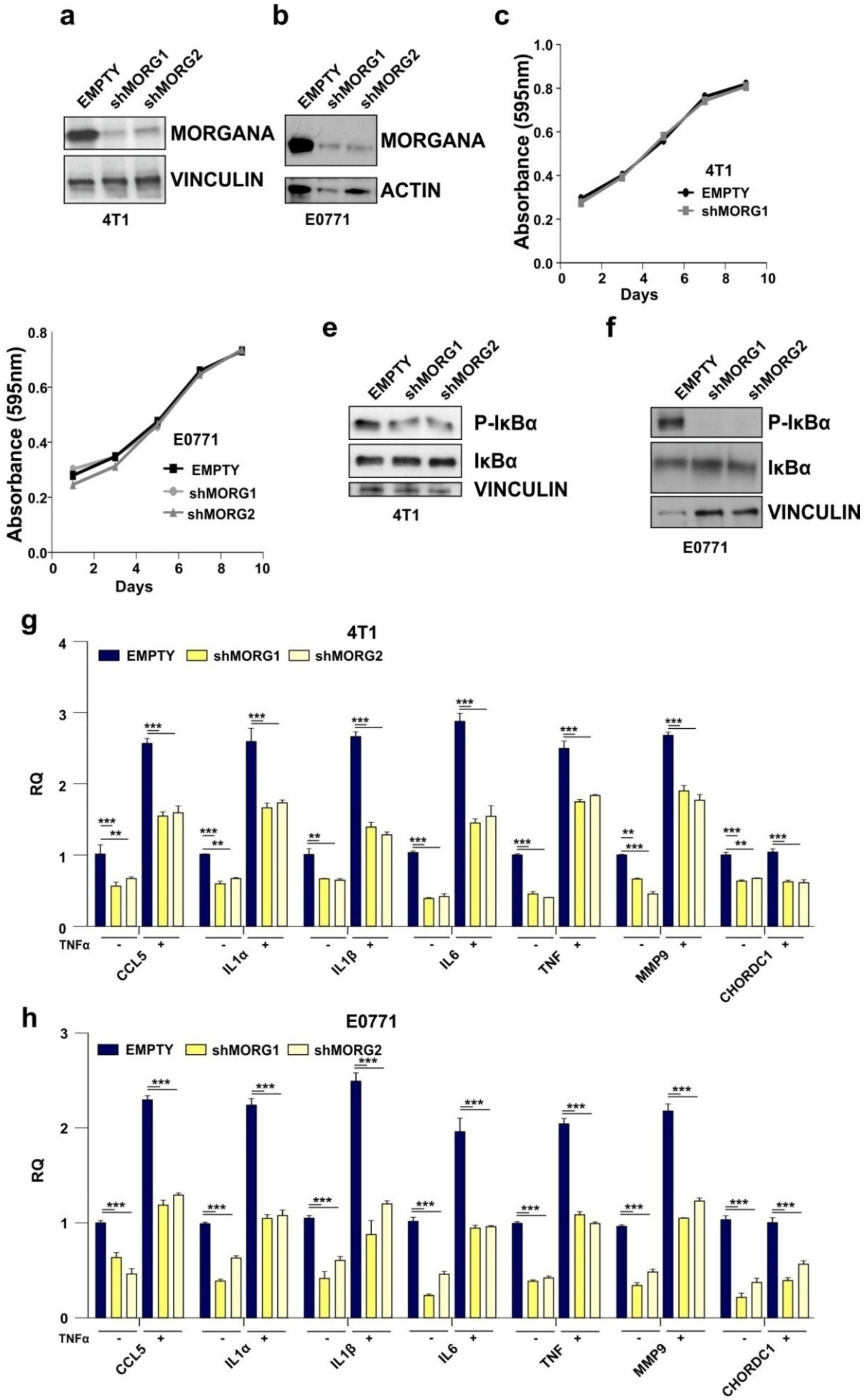

Supplementary Figure 8. NF-κB activation is regulated by Morgana levels in mouse breast cancer cells. (**a**) Immunoblot of 4T1 infected with empty vector (EMPTY) or two different morgana shRNAs (shMORG1 and shMORG2). (**b**) Immunoblot of E0771 EMPTY or shMORG1 and shMORG2. (**c,d**) Growth curves of 4T1 (**c**) and E0771 (**d**) EMPTY or shMORG1 and shMORG2. (**e,f**) Western blot analysis of P-IκBα, IκBα and Vinculin in 4T1 (**e**) and E0771 (**f**) EMPTY or shMORG1 and shMORG2. (**g,h**) Gene expression analysis by Real-time PCR of NF-κB target genes in 4T1 (**g**) and E0771 (**h**) EMPTY or shMORG1 and shMORG2 treated or not with 10nM TNFα for 4h. Data are the results of three independent experiments. Bars in graphs represent standard errors (**$p < 0.01$; ***$p < 0.001$).

Supplementary Figure 9

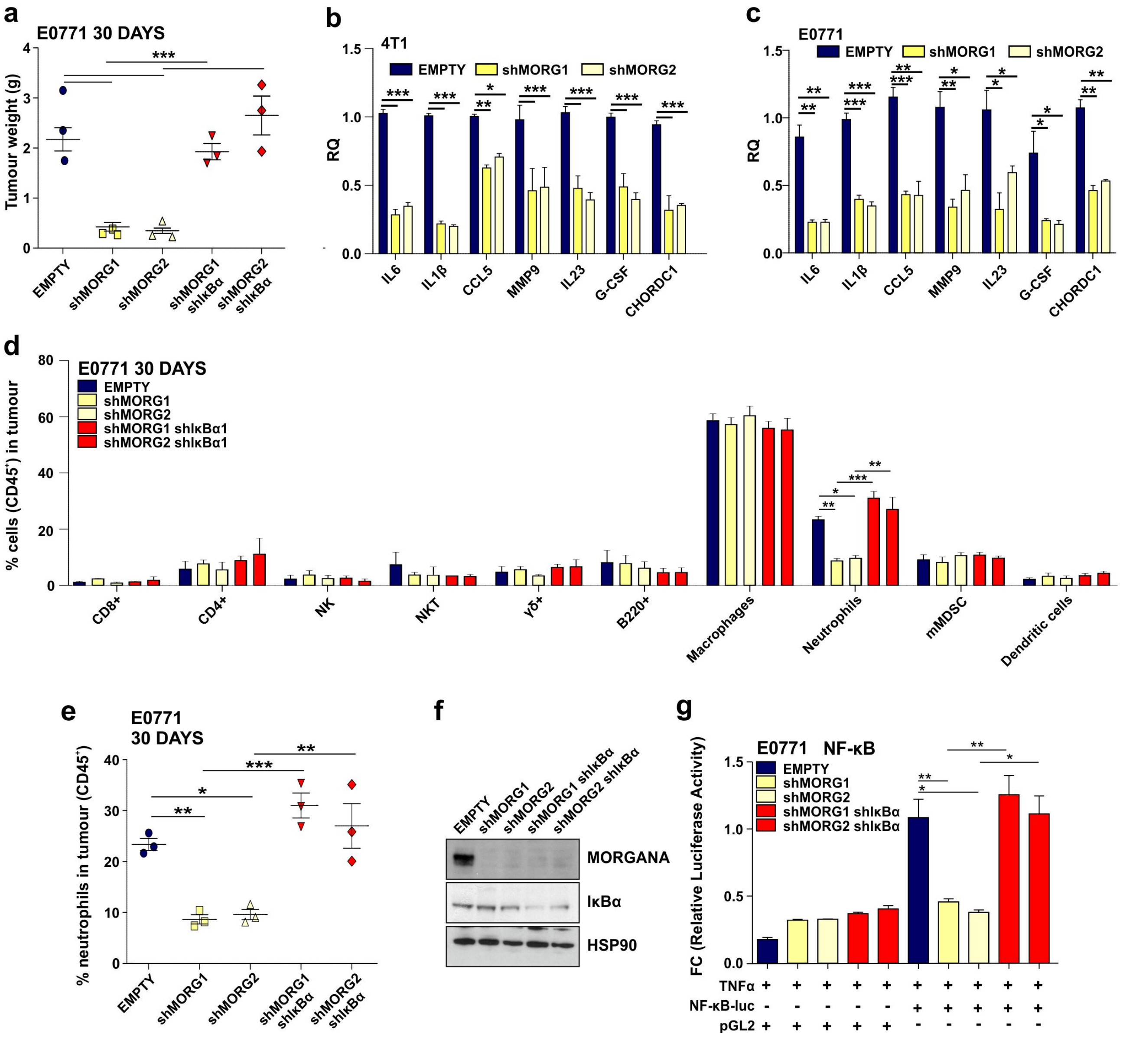

Supplementary Figure 9. Morgana-NF-κB axis in breast cancer cells induces neutrophil recruitment in primary tumour. (**a**) Tumour weight 30 days after subcutaneous injection of E0771 cells EMPTY or shMORG1 and shMORG2, infected or not with IκBα shRNA (*n*=3 C57BL/6 mice per group). (**b**,**c**) Gene expression analysis by Real-time PCR of NF-κB target genes analyzed in primary tumours derived from 4T1 (**b**) or E0771 (**c**) EMPTY or shMORG1 and shMORG2. (**d**) Percentage of immune cells (gated on $CD45^+$ cells) detected in the primary tumour 30 days after injection of E0771 cells described in **a** (*n*=3 C57BL/6 mice per group). (**e**) Percentage of neutrophils detected in the primary tumour of each mouse 30 days after injection of E0771 described in **a**. (**f**) Immunoblotting of Morgana, IκBα and HSP90 in E0771 cells described in **a**. (**g**) Luciferase assays of NF-κB activity in E0771 cells described in **a** transfected with a NF-κB luciferase reporter or control vector. Bars in graphs represent standard errors (*p < 0.05; **p < 0.01; ***p < 0.001).

Supplementary Figure 10

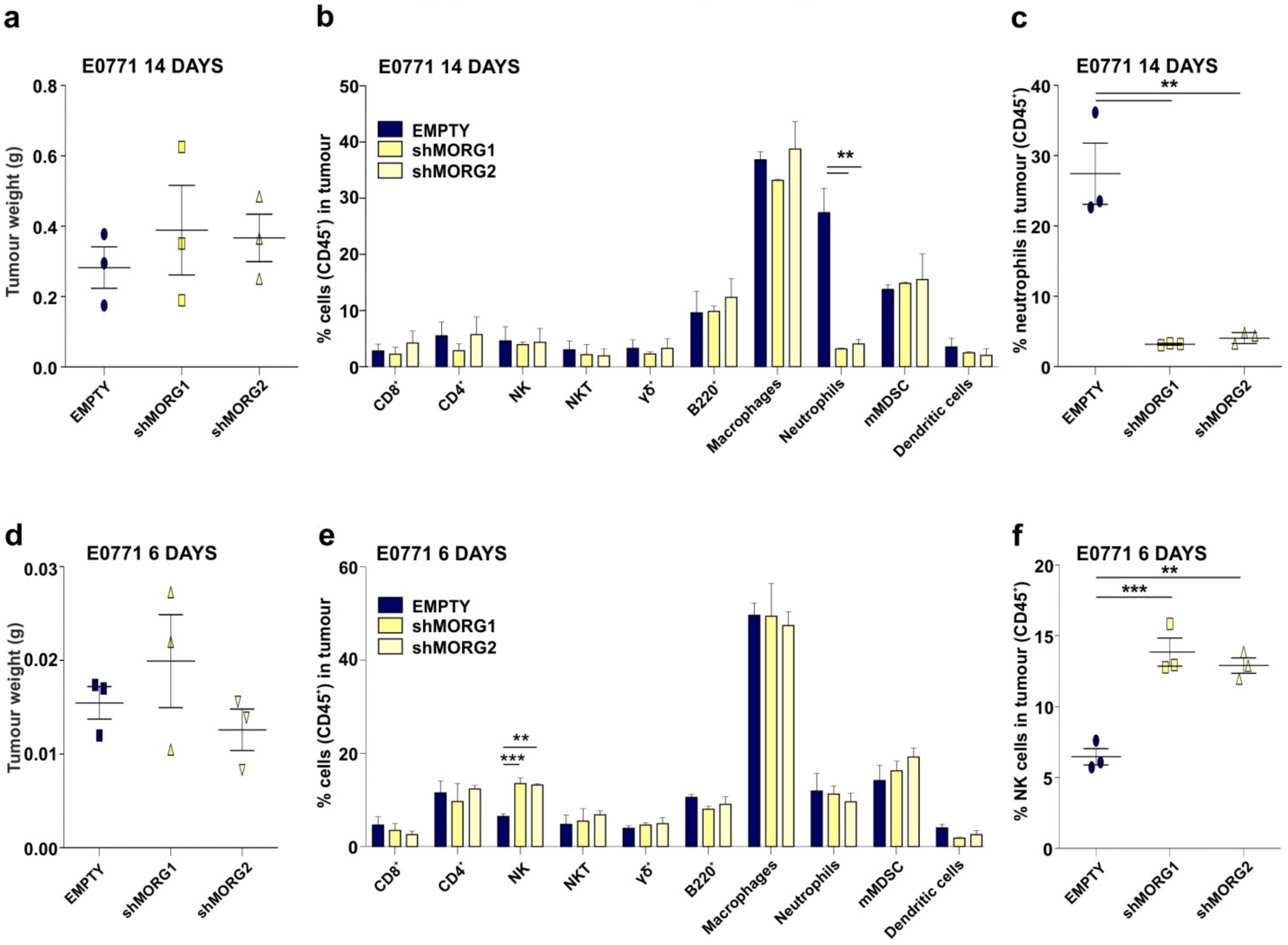

Supplementary Figure 10. High Morgana expression levels in breast cancer cells induces neutrophil and natural killer (NK) cells recruitment in primary tumour. (**a**) Tumour weight 14 days after subcutaneous injection of E0771 cells EMPTY or shMORG1 and shMORG2 (*n*=3 C57BL/6 mice per group). (**b**) Percentage of immune cells (gated on $CD45^+$ cells) detected in the primary tumour 14 days after injection of E0771 EMPTY or shMORG1 and shMORG2 (*n*=3 C57BL/6 mice per group). (**c**) Percentage of neutrophils detected in the primary tumour of each mouse 14 days after injection of E0771. (**d**) Tumour weight 6 days after subcutaneous injection of E0771 cells EMPTY or shMORG1 and shMORG2 (*n*=3 C57BL/6 mice per group). (**e**) Percentage of immune cells (gated on $CD45^+$ cells) detected in the primary tumour 6 days after injection of E0771 EMPTY or shMORG1 and shMORG2 (*n*=3 C57BL/6 mice per group). (**f**) Percentage of NK cells detected in the primary tumour of each mouse 6 days after injection of E0771. Bars in graphs represent standard errors (**$p < 0.01$; ***$p < 0.001$).

Supplementary Figure 11

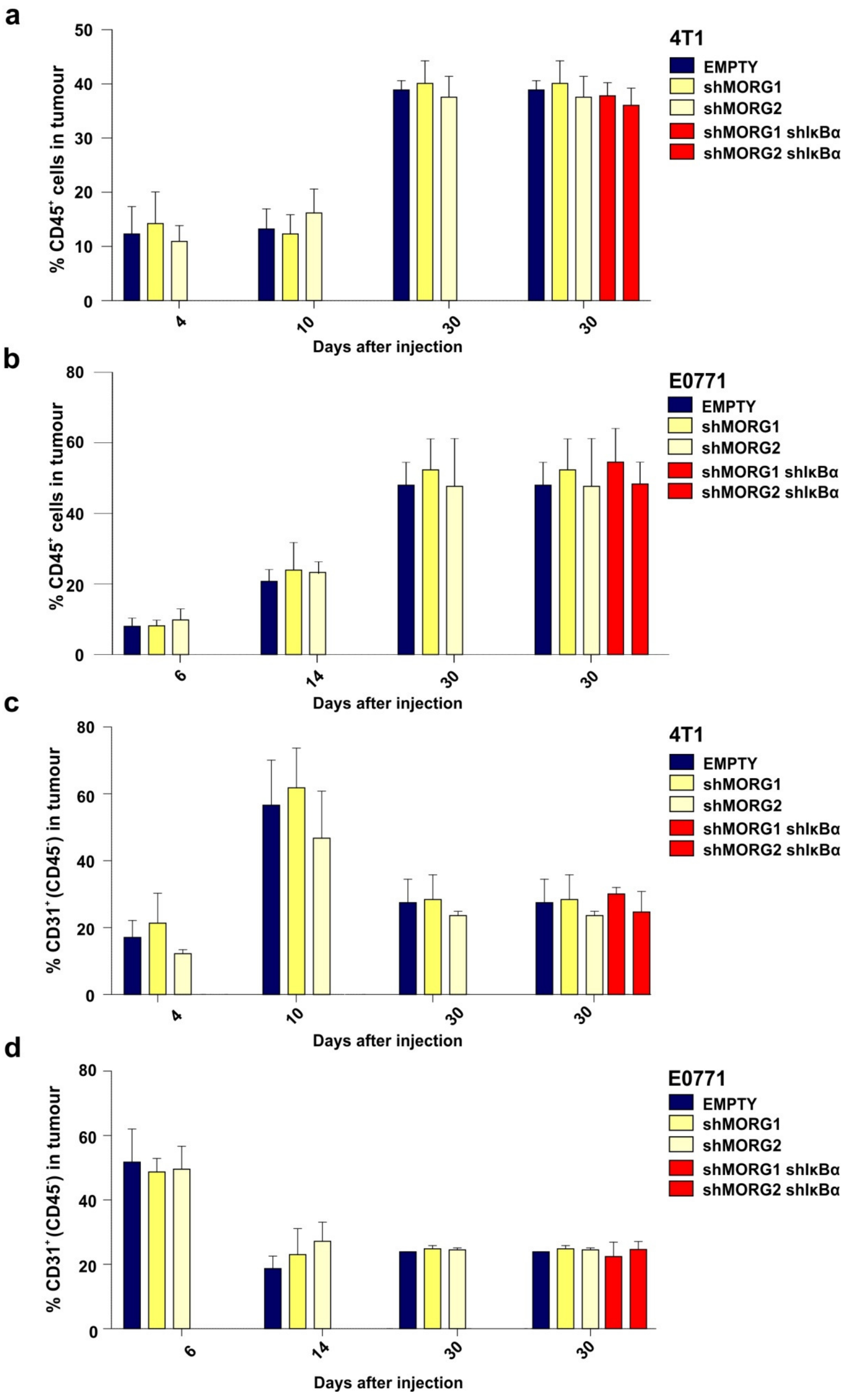

Supplementary Figure 11. Variations in Morgana expression levels in breast cancer cells does not affect CD45$^+$ and endothelial (CD31$^+$) cells recruitment in primary tumour. (**a**) Percentage of CD45$^+$ cells detected in the primary tumour at different time points analyzed after subcutaneous injection of 4T1 EMPTY or shMORG1 and shMORG2 (*n*=3 BALB/c mice per group). (**b**) Percentage of CD45$^+$ cells detected in the primary tumour at different time points analyzed after subcutaneous injection of E0771 EMPTY or shMORG1 and shMORG2 (*n*=3 C57BL/6 mice per group). (**c**) Percentage of CD31$^+$ cells (gated on CD45$^-$ cells) detected in the primary tumour at different time points analyzed after injection of 4T1 EMPTY or shMORG1 and shMORG2 (*n*=3 BALB/c mice per group). (**d**) Percentage of CD31$^+$ cells (gated on CD45$^-$ cells) detected in the primary tumour at different time points analyzed after subcutaneous injection of E0771 EMPTY or shMORG1 and shMORG2 (*n*=3 C57BL/6 mice per group).

Supplementary Figure 12

Figure 4a

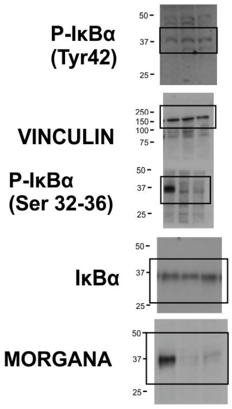

Figure 4c

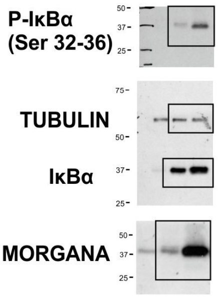

Figure 4d

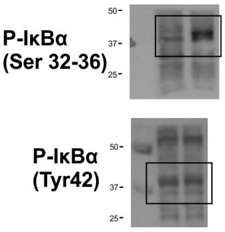

Figure 4e

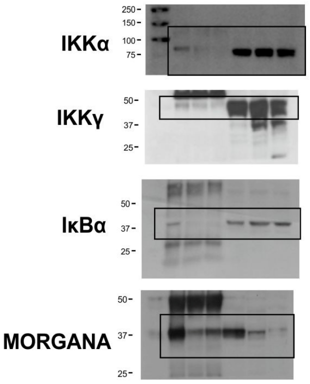

Figure 4f

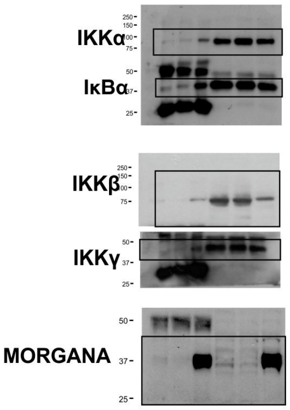

Figure 4g

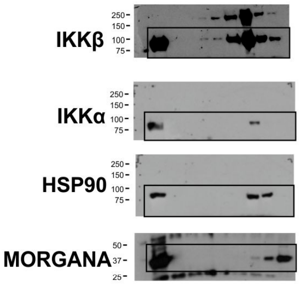

Figure 4h

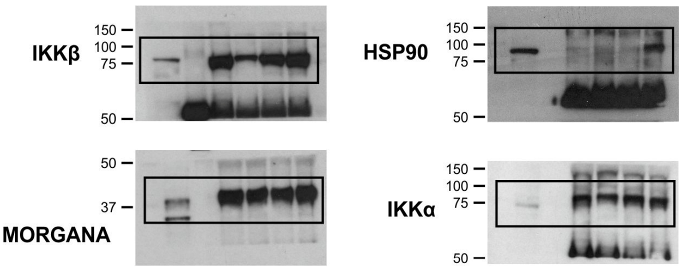

Figure 4i

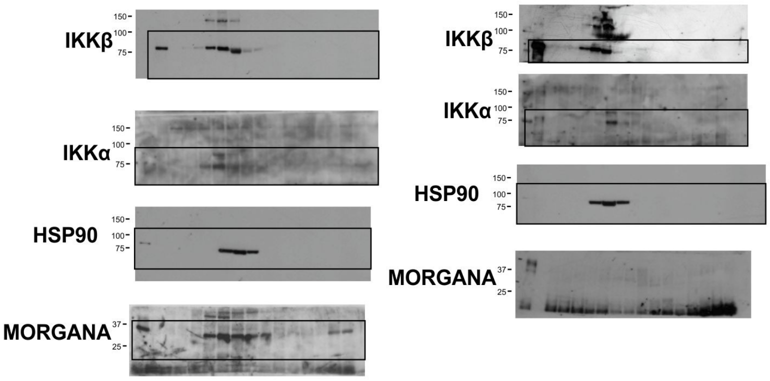

Figure 4k

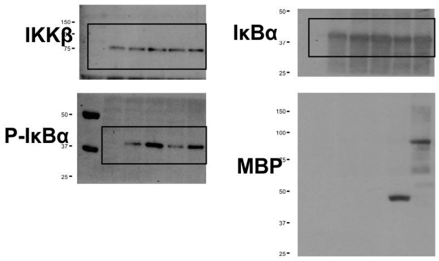

Figure 4j

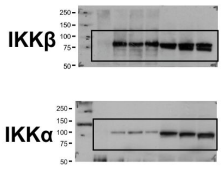

Figure 4l

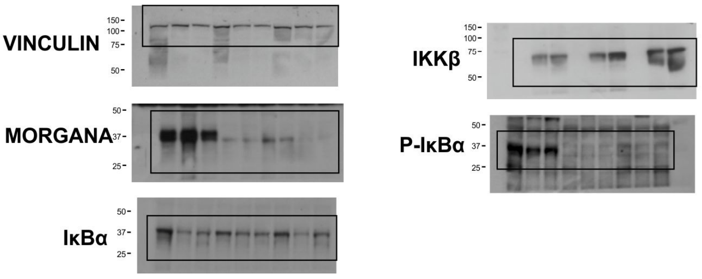

Supplementary Figure 12. Uncropped images of the most important Western blot figures shown in the manuscript.

Supplementary Tables

Supplementary Table 1. Gene Ontology enrichment on microarray data.

GO id	Term	Ontology	common	expected	p_value
GO:0051240	MHC protein complex	CC	19	0.7032	3,67E-22
GO:0019221	cytokine-mediated signaling pathway	BP	57	13.61	2,85E-17
GO:0071345	cellular response to cytokine stimulus	BP	62	16.93	5,32E-16
GO:0042613	MHC class II protein complex	CC	13	0.4219	5,89E-16
GO:0034097	response to cytokine	BP	65	19.58	1.07e-17
GO:0071556	integral component of lumenal side of endoplasmic reticulum membrane	CC	15	0.7032	1,12E-14
GO:0098576	lumenal side of membrane	CC	15	0.7314	2.57e-17
GO:0034341	response to interferon-gamma	BP	28	3.657	2,72E-14
GO:0006952	defense response	BP	102	42.59	3,78E-14

Supplementary Table 1. Table showing the top 10 GO terms by enrichment P-value among the down-regulated genes obtained by microarray analysis

Supplementary Table 2. GSEA analysis on microarray data.

Gene sets related to NFKB	p.geomean	stat.mean	p.val	q.val	set.size	exp1
HALLMARK TNFA SIGNALING VIA NFKB	0,0020	-2,8987	0,0020	0,0660	194,0000	0,0020
NFKB1 TARGETS (RASHI)	0,0122	-2,3713	0,0122	0,1608	19,0000	0,0122
NFKB TARGETS KERATINOCYTE UP (HINATA)	0,0174	-2,1330	0,0174	0,1608	85,0000	0,0174
GGGNNTTTCC V$NFKB Q6 01	0,0222	-2,0215	0,0222	0,1608	119,0000	0,0222
V$NFKB C	0,0244	-1,9766	0,0244	0,1608	228,0000	0,0244
CORE NFKB PATHWAY (GILMORE)	0,0379	-1,8542	0,0379	0,1655	14,0000	0,0379
V$NFKB Q6 01	0,0407	-1,7469	0,0407	0,1655	208,0000	0,0407
NFKB IMMU INF (HINATA)	0,0418	-1,8084	0,0418	0,1655	17,0000	0,0418
NFKB SIGNALING (SCHOEN)	0,0451	-1,7305	0,0451	0,1655	33,0000	0,0451

Supplementary Table 2. Table showing NF-κB related gene sets positively correlated with CHORDC1 mRNA levels generated by GSEA analysis of microarray data obtained from MDA-MB-231 EMPTY versus shMORG1.

Supplementary Table 3. Correlation in mRNA levels between Morgana and immune system cell markers.

GENE	Rho	Pvalue
**FCGR3B**	0.1331	9.81E-06
**FCGR3A**	0.1302	1.56E-02
**CD68**	0.03826	0.2059
**CD14**	0.04337	0.1516
**CD19**	-0.03843	0.2039
**MS4A1**	0.03071	0.31

Supplementary Table 3. Spearman correlation in mRNA levels between CHORDC1 (Morgana coding gene) and neutrophil markers (FCGR3A and FCGR3B) or macrophage markers (CD68 and CD14) or lymphocyte markers (CD19 and MS4A1).

Supplementary Table 4: primers used for real time PCR

Primer	Forward primer (5'-3')	Reverse primer (5'-3')	Probe
**MMP9 HS**	gaaccaatctcaccgacagg	gccacccgagtgtaaccata	6
**MMP2 HS**	agaaggctgtgttctttgcag	aggctggtcagtggcttg	1
**CHORDC1 (MORGANA) HS**	gccagtagaagcaataaaaagacc	tgatgacagtttaagtttatcaagtgc	16
**CCL5 HS**	tgcccacatcaaggagtattt	ctttcgggtgacaaagacg	59
**IL1A HS**	ggttgagtttaagccaatcca	tgctgacctaggcttgatga	6
**IL1B HS**	tacctgtcctgcgtgttgaa	tctttgggtaatttttgggatct	78
**IL6 HS**	gatgagtacaaaagtcctgatcca	ctgcagccactggttctgt	40
**TNF HS**	cagcctcttctccttcctgat	gccagagggctgattagaga	29
**CCL5 MM**	tgcagaggactctgagacagc	gagtggtgtccgagccata	110
**IL1A MM**	ttggttaaatgacctgcaaca	gagcgctcacgaacagttg	52
**IL1B MM**	agttgacggaccccaaaag	agctggatgctctcatcagg	38
**TNF MM**	ctgtagcccacgtcgtagc	ttgagatccatgccgttg	25
**MMP2 MM**	agaaggctgtgttctttgcag	aggctggtcagtggcttg	2
**MMP9 MM**	acgacatagacggcatcca	gctgtggttcagttgtggtg	19
**CHORDC1 (MORGANA) MM**	gaagcaataaaaaggccaagc	tcactcccagatgacagtttaagt	38
**IL6 MM**	gctaccaaactggatataatcagga	ccaggtagctatggtactccagaa	6
**IL17A MM**	cagggagagcttcatctgtgt	gctgagctttgagggatgat	74
**CSF3 (G-CSF) MM**	gctgctggagcagttgtg	gggatccccagagagtgg	17
**IL23 MM**	tccctactaggactcagccaac	agaactcaggctgggcatc	19
**GM7030 (HLA-F) MM**	gcacatgcgtggagacac	cgggtcacatgtgtatttgg	69
**HLA-B HS**	cctacctggagggcgagt	ggtgggtcacgtgtgtcttt	80
**HLA-F HS**	tcagatagaaacagagggagctact	agcccaaatatccttggaaga	2
**CXCL1 MM**	agactccagccacactccaa	tgacagcgcagctcattg	83
**CXCL2 MM**	aaaatcatccaaaagatactgaacaa	ctttggttcttccgttgagg	26
**CXCL5 MM**	tagagccccaatctccacac	ggagctggaggctcattgt	67
**CXCL12 MM**	ccaaactgtgcccttcagat	atttcgggtcaatgcacact	41
**FIBRONECTIN MM**	gaagcaataaaaaggccaagc	gtagggcttttcccaggtct	3
**LOX MM**	caggctgcacaatttcacc	caaacaccaggtacggcttt	48
**S100A8 MM**	tccttgcgatggtgataaaa	ggccagaagctctgctactc	5
**S100A9 MM**	caccctgagcaagaaggaat	tgtcatttatgagggcttcattt	31

Supplementary Table 5: list of antibodies used for flow cytometry

**Name**	**Company**	**Clone/#**	**Dilution**
CD11b-FITC and APC	Biolegend	M1/70	(1:100)
F4/80-FITC	Biolegend	BM8	(1:100)
CD206 -PE	Biolegend	C068C2	(1:100)
CD3ε - FITC	Biolegend	145-2C11	(1:100)
CD8 - VioBlue	Milteny	53-6.7	(1:50)
CD4 - APCCy7	Biolegend	Gk1.5	(1:100)
Ly6G- VioBlue	Milteny	1A8	(1:50)
CD49b PE	Milteny	DX5	1:50
B220 PECY7	Biolegend	RA3-6B2	1:100
CD45 VioGreen	Milteny	30F11	1:50
TCRγδ APC	Milteny	GL3	1:50
CD31 PECY7	Biolegend	390	1:100
Ly6C-APCCY7	Biolegend	HK1.4	1:100
CD64 PE	Biolegend	X54-5/7.1	1:100
CD24 PECY7	Biolegend	M1/69	1:100
MHCII APC	Milteny	M5/114.15.2	1:50
CD11C APCCY7	Biolegend	N418	1:100
Annexin V FITC	Biolegend		(1:200)
